# Sarcopenia prevalence and association with nutritional status in cohort of elderly patients affected by musculoskeletal concerns: a real-life analysis

**DOI:** 10.3389/fendo.2023.1194676

**Published:** 2023-06-26

**Authors:** Maria Chiara Maccarone, Daniele Coraci, Andrea Bernini, Nicola Sarandria, Marta Rossella Valente, Anna Chiara Frigo, Yannis Dionyssiotis, Stefano Masiero

**Affiliations:** ^1^ Physical Medicine and Rehabilitation School, Department of Neuroscience, University of Padova, Padua, Italy; ^2^ Department of Neuroscience, Rehabilitation Unit, University of Padova, Padua, Italy; ^3^ Biostatistics, Epidemiology and Public Health Unit, Department of Cardiac, Thoracic and Vascular Sciences, University of Padova, Padua, Italy; ^4^ Spinal Cord Injury Rehabilitation Clinic, University of Patras, Patras, Greece

**Keywords:** early diagnosis, frailty, sarcopenic obesity, rehabilitation, frailty prevention, adult, aged

## Abstract

**Introduction:**

The progressive loss of skeletal muscle mass, strength, and function that frequently occurs as people get older is referred to as sarcopenia. Elderly musculoskeletal aging, sarcopenia, and obesity are all intimately connected. Our study’s aim is to investigate the prevalence of sarcopenia in a real cohort of patients over 65 with musculoskeletal conditions referring to a Rehabilitation Unit. The secondary aim of our study is to investigate associations between sarcopenia and alterations in nutritional status and Body Mass Index (BMI). Finally, quality of life and global health has been investigated in our population.

**Materials and methods:**

From January 2019 to January 2021, 247 patients over 65 years old with musculoskeletal concerns were enrolled and participated in an observational study. As outcome measures, the Mini Nutritional Assessment (MNA), the 12-Item Short Form Health Survey (SF-12), and the Cumulative Illness Rating Scale Severity Index (CIRS-SI) were used. Additionally, measurements of total skeletal muscle mass (SMM) and appendicular muscle mass (ASMM) using bioelectrical impedance analysis, as well as a hand grip strength test of the non-dominant hand were taken. The Mid Upper Arm Circumference (MUAC) and the Calf Circumference (CC) were measured and recorded as further indications of possible sarcopenia.

**Results:**

A percentage of 46.1% of subjects with overt sarcopenia was found and 10.1% showed a severe sarcopenia. Patients with severe sarcopenia showed significantly lower values of BMI and MNA. Additionally, sarcopenic patients showed significantly lower values in MNA when compared to non-sarcopenic patients. Considering SF-12, only the physical score revealed slight significant differences. In particular, patients affected by probable or severe sarcopenia presented a lower value than non-sarcopenic patients. Concerning MUAC and CC, severe sarcopenic patients showed significant lower values for both the body parts.

**Conclusion:**

Our study considers a cohort of real-life elderly subjects with musculoskeletal concerns and shows that these subjects are highly susceptible to sarcopenia. Therefore, rehabilitation for elderly patients with musculoskeletal concerns requires to be customized and multidisciplinary. Future research should further investigate these aspects in order to enable the early identification of sarcopenia and the formulation of customized rehabilitative programs.

## Introduction

According to the current definition, sarcopenia refers to the progressive loss of skeletal muscle mass, strength and performance that increases risk of physical disability and hospitalization (1). Sarcopenia is believed to be a condition typical of advanced age, even if muscle mass loss begins around the age of 40 (1, 2). Nevertheless, while muscle mass represents the 42% of global body mass in adults, it drops to about 27% in the elderly (3). Sarcopenia’s negative effects can affect older subjects with a prevalence varying from 10% to 27% (4), increasing the risk of adverse consequences such as falls, fractures, depression, physical impairment, quality of life worsening, and increased mortality (5–8).

Sarcopenia pathophysiology is complex, with aging, sociodemographic factors, lifestyle choices, and a number of medical conditions being all factors that contribute (2). In particular, musculoskeletal aging and sarcopenia in the elderly have been demonstrated to be closely linked, since numerous studies have shown that cellular, mitochondrial and nervous impairment underlying ageing dysfunctions can also lead to the appearance of sarcopenia (9, 10). On the other hand, the adipose tissue redistribution represents another important age-related effect. In fact, as people age, subcutaneous adipose tissue declines gradually, visceral obesity increases, and adipocytes and lipids accumulate in the bone marrow, liver, and skeletal muscle (myosteatosis). In particular, total body fat increases with age until it reaches a plateau, and then it gradually begins to decline. Obesity and excessive caloric consumption can both contribute to the development of sarcopenia (11–13). Sarcopenia obesity supervenes when a decrease of lean body mass accompanied by an excessive accumulation of adipose tissue, particularly visceral fat, occurs (3, 14).

Even if musculoskeletal concerns are among the most common causes for older people to be admitted to a rehabilitation unit, to date, there are no studies that have investigated in real life the incidence of sarcopenia among elderly patients with musculoskeletal disorders. Therefore, our study aims to investigate the prevalence of sarcopenia (diagnosed through the algorithm proposed by the European Working Group on Sarcopenia in Older People-2 or EWGSOP-2) in a real-life cohort of patients over the age of 65 with musculoskeletal conditions referred to a Rehabilitation Unit on an outpatient basis. Given the association between sarcopenia and obesity, secondary aim of our study is to search for real-life associations between sarcopenia and changes in nutritional status and Body Mass Index (BMI) among a population of elderly subjects suffering from musculoskeletal concerns. Finally, quality of life and global health has been investigated in our population.

## Materials and methods

### Study design

An observational, prospective study was conducted involving a cohort of 247 patients with musculoskeletal disorders, enrolled from January 2019 to January 2021 at the Rehabilitation Unit of Padua University – General Hospital, Padua, Italy.

### Participants

Subjects of both sexes over the age of 65 who had a diagnosis of musculoskeletal disorders were included in the study. The population under study included only community-dwelling subjects. Patients involved in the study presented a diagnosis of a musculoskeletal disorder, e.g., osteoarthritis, shoulder tendonitis, and chronic back pain. For the diagnosis of osteoarthritis, the diagnostic criteria were based on the Kellgren-Lawrence grading system for radiographic assessment, while patients with post-bone fracture outcomes and chronic back pain had a well-established diagnosis made by a physician (medical history, clinical examination, imaging studies and surgical reports.). Enrolled subjects must be able to provide informed consent. In addition, enrolled patients had to complete the Short Portable Mental Status Questionnaire (SPMSQ), reporting a score ≥ 5, be able to walk without aids, and not present any conditions that would prevent them from perform bioelectrical impedance analysis (BIA).

People less than 65 years of age, non-community dwelling (nursing home, institutional setting, etc.) were excluded. Heart failure, respiratory failure, impaired cognitive functions (mini mental status examination<24), multiple musculoskeletal conditions, oncological or psychiatric comorbidities, and inability to properly comprehend and sign informed consent represented the exclusion criteria. Moreover, as an exclusion criterion, participants with specific diagnoses (such as spinal fractures, tumors, bone infections) that could potentially confound the study outcomes were excluded.

### Outcome measures

Three different evaluation scales in the Italian validated version were employed:

- The Cumulative Illness Rating Scale Severity Index (CIRS-SI): a standardized instrument used in the geriatric field to measure the health of the elderly as objectively as possible. It requires the physician to assess and measure the clinical and functional severity of 14 disease categories. For each of these categories, a severity value must be defined, based on clinical history, objective examination and patient-reported symptoms. The scale provides a cumulative score, which can range from 0 to 56 (15).
- The Mini Nutritional Assessment (MNA): a screening tool that contribute to identify elderly patients who are malnourished or at risk of malnutrition. Thanks to 18 questions grouped in 4 sections (anthropometry, general state, eating habits and self-perceived health and nutrition states), the MNA provides a multidimensional assessment of the patient nutritional condition. The final score can reach a maximum of 30 points and allows the nutritional status to be classified: patients are considered well-fed when they reach a score ≥24 points, while they are at risk of malnutrition with a score between 23.5 and 17 (16).
- The 12-Item Short Form Health Survey (SF-12): a questionnaire readjusted from a larger version, the 36-Item Short Form Health Survey (SF-36), used to investigate the perception of personal psychophysical conditions, frequently employed in the rehabilitation field. The SF- 12 results, dual and expressed by the acronyms PCS (Physical Component Summary) and MCS (Mental Component Summary), can adequately summarize the size of the patient’s impairment both from a physical and mental point of view (17).

Instrumental evaluations were also carried out, including:

- Hand grip strength evaluation of the non-dominant hand measured with a handheld dynamometer.
- Total Skeletal Muscular Mass (SMM) and Appendicular Skeletal Muscle Mass (ASMM) measurement through a bioelectrical impedance analysis employed to assess body composition (Biody Xpert, Interfit technology) (18).

The estimation of muscular masses, using the bioelectrical impedance analysis is well accepted. They are based on equation that combine height, sex, age and the parameters calculated by the proper instrument:


SMM (kg)={(Height ^2/R·0.401) + (sex·3.825) + [age·(−0.071)]} +5.102

$$\begin{array}{c} ASMM\;(kg)=-3.964\;+\;(0.227\cdot RI)\;+\;(0.095\cdot Weight)\;+\;(1.384\cdot sex)\;\\ +\;(0.064\cdot Xc) \end{array}$$

where height is in cm, R is the resistance in Ohms, RI the resistance normalized for the height, the sex is 0 for biological females and 1 for biological males, age is in years and the Xc is the reactance measured in Ohms (19, 20).

Sarcopenia was considered probable when low muscle strength was detected. Sarcopenia diagnosis was confirmed if also low muscle quantity or quality was recorded. When low muscle strength, low muscle quantity/quality and low physical performance were all detected, sarcopenia was considered severe. In particular, according to the European Working Group on Sarcopenia in Older People 2 (EWGSOP2), the following values were used to define sarcopenia cut-off (21):

- Hand Grip Strength< 27 kg for male subjects and< 16 kg for female subjects;
- ASMM< 20 kg for male subjects and< 15 kg for female subjects;
- ASMM/height^2^< 7.0 kg/m^2 for male subjects and< 6.0 kg/m^2 for female subjects.

According to the EWGSOP2, severe sarcopenia was defined testing gait speed through the 4 meters walking test: a gait speed ≤0.8 m/s both for males and females allowed to define a severe form of sarcopenia. The Calf Circumference (CC) and the Mid Upper Arm Circumference (MUAC) were measured and collected as further indications of possible sarcopenia, according to Hu et al. for MUAC (22) and Kawakami et al. for CC (23).

### Statistical analysis

For the statistical analysis, we divided the whole sample of patients into four groups, according to the sarcopenia level measured by the association of strength, speed and ASMM/height^2^ (levels: 0, normal; 1, probable sarcopenia, only reduced muscle strength; 2, sarcopenia, reduced strength and muscle mass; 3, severe sarcopenia, reduced strength, muscle mass and performance). To show the distribution of the sex and the CIRS-SI in relation to the sarcopenia level, we used a contingency table.

In order to evaluate the difference in BMI, MNA, SF-12 and arm and calf circumference among the four groups, we used the Kruskal-Wallis test, applying the Bonferroni correction for the repetitive measures. This test was used for the comparison of independent variables of multiple groups. Finally, Spearman correlation was employed to evaluate the association of the arm and calf circumference with other variables. We decided to use non-parametric tests because of the features of the data.

Quantitative data were shown as median values. The analyses were performed using the IBM SPSS Statistics Software version 26 and the significance was set as p< 0.05.

## Results

A total of 247 patients were enrolled in our study, with a median age of 73 years (range 61 – 95 years). Among them, 98 men and 149 women were studied (**Table 1**). A percentage of 46.1% of subjects with overt sarcopenia was found and 10.1% showed a severe sarcopenia. The majority of the subjects presented a sarcopenia level 2 and low levels of global health, as assessed by the CIRS-SI (**Table 2**).

**Table 1:** Data of age, BMI and anthropometric measures related to sex.

Sex	Number of patients	Age	BMI	MUAC	CC
Female	98	72.8	26.3	28.5	36.8
Male	149	73.4	26.7	28.2	35.8
Total	247	73.1	26.5	28.3	36.2

BMI, Body Mass Index; MUAC, Mid Upper Arm Circumference; CC, Calf Circumference.

**Table 2:** Data of MNA and SF-12 related to sarcopenia level.

Sarcopenia level	Number of patients	Percentage	MNA	SF-12 (PCS)	SF-12 (MCS)
0	64	25.9%	26.8	13.9	18.2
1	69	27.9%	25.7	12.7	17.5
2	89	36.1%	25.4	13.2	17.9
3	25	10.1%	23.2	11.7	17.3
Total	247	100.00%	25.6	13.1	17.8

Level 0, no sarcopenia; level 1, probable sarcopenia; level 2, sarcopenia; level 3, severe sarcopenia; MNA, Mini Nutritional Assessment; SF-12 (PCS), 12-Item Short Form Health Survey Physical Component Summary; SF-12 (MCS), 12-Item Short Form Health Survey Mental Component Summary.

**Figure 1:**
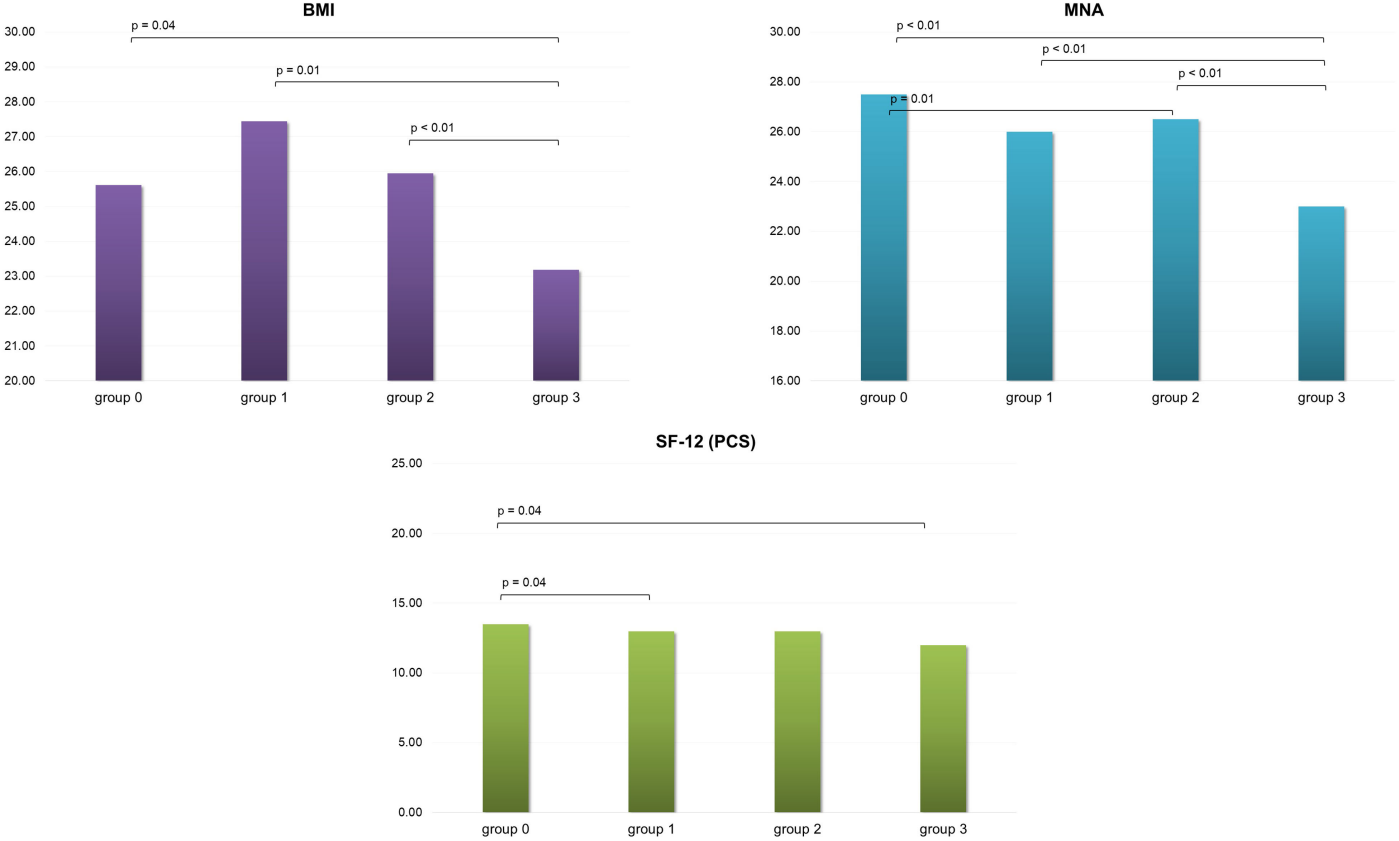
BMI, MNA and SF-12 (PCS) scores variation in the four group of patients evaluated.BMI, Body Mass Index; MNA, Mini Nutritional Assessment; SF-12 (PCS), 12-Item Short Form Health Survey Physical Component Summary; group 0, no sarcopenia; group 1, probable sarcopenia; group 2, sarcopenia; group 3, severe sarcopenia.

Considering the different sarcopenia levels, the most severe level group (level 3) showed significantly lower values of BMI and MNA in comparison with all the other groups. Additionally, level 2 showed significant lower values in MNA, exclusively in comparison with level 0. Considering SF-12, only the physical composite score revealed slight significant differences. In particular, group 3 and group 1 presented lower values than group 0 (**Figure 1**).

**Figure 2:**
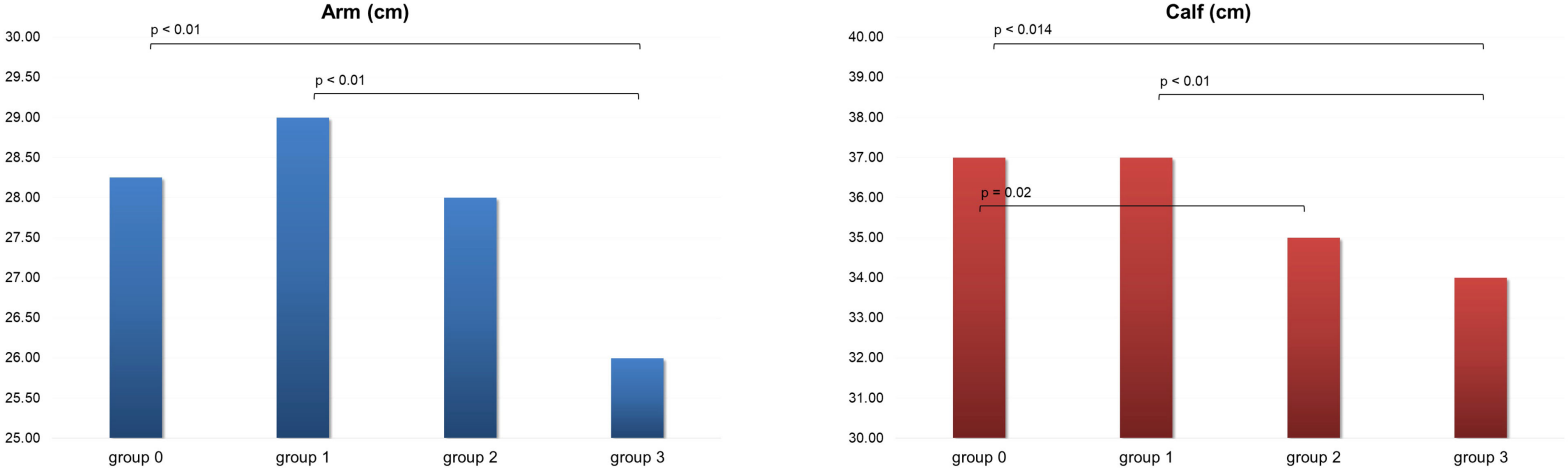
Arm and calf circumference in the four group of patients evaluated. Group 0, no sarcopenia; group 1, probable sarcopenia; group 2, sarcopenia; group 3, severe sarcopenia.

**Figure 3:**
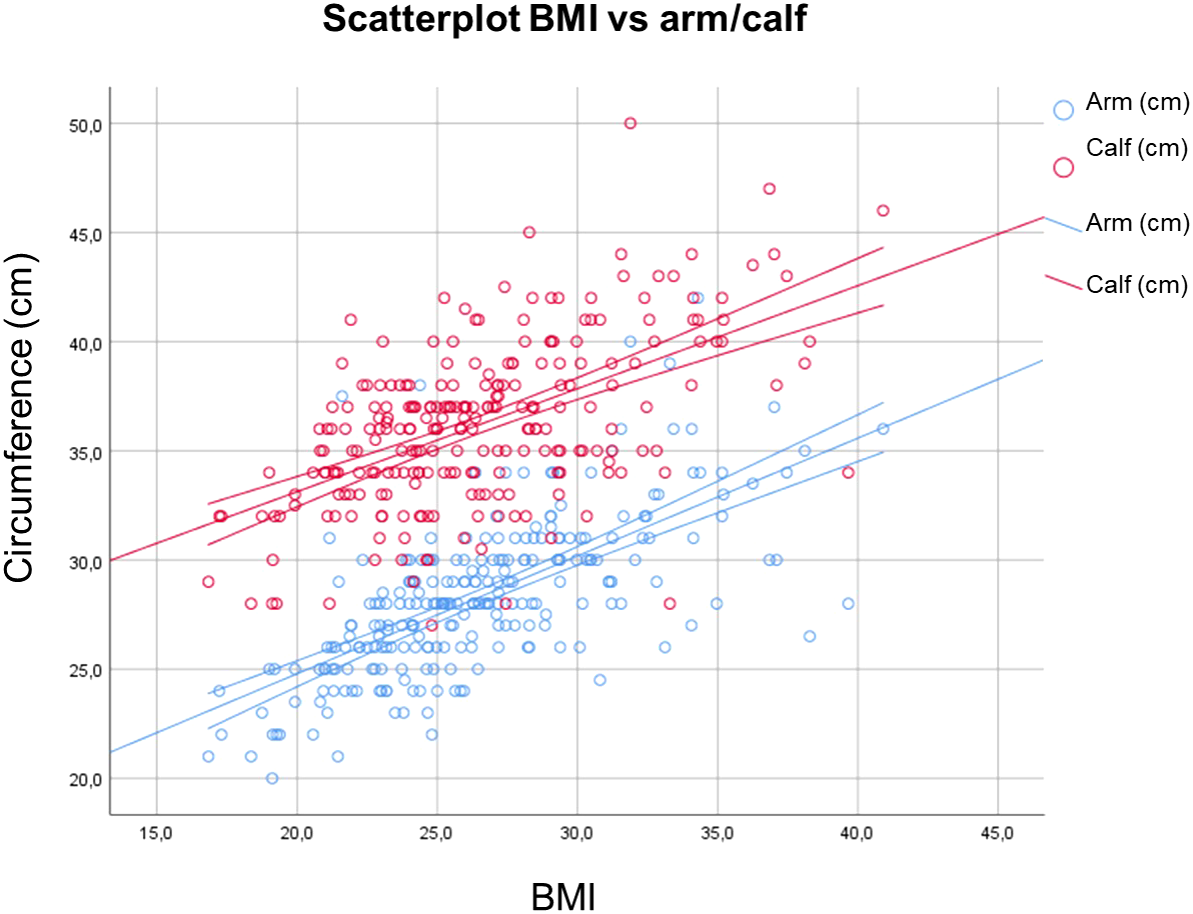
Correlations between BMI and MUAC and BMI and CC. BMI, Body Mass Index; MUAC, Mid Upper Arm Circumference; CC, Calf Circumference.

Concerning the arm and calf circumference, group 3 showed significant lower values for both the body parts in comparison with groups 0 and 1. Sarcopenia level 2 showed lower values than group 0 in CC (**Figure 2**). Significant direct correlations were found between BMI and MUAC and BMI and CC (p< 0.01, r = 0.70 and r = 0.52 respectively). A minimal direct correlation was found between MNA and CC (**Figure 3**).

## Discussion

In our study, elderly patients with musculoskeletal disorders who accessed the outpatient clinics of the University of Padua Rehabilitation Unit were shown to frequently present sarcopenia and low levels of global health, as assessed by the CIRS-SI score. In particular, the sarcopenia prevalence in our study reconfirmed data already present in the literature (24). In our work, we decided to apply ASMM because it is reliable and shows a relative cost-effectiveness. The application of the parameters calculated by the bioelectrical impedance and the use of SMM and ASMM allowed us a clear identification of the four groups, with different level of sarcopenia. The groups showed some peculiar differences and let us speculate about patients’ conditions and the different approaches to management.

Interestingly, when measuring BMI in patients referred to our center, we found that patients with severe sarcopenia had a sharp decline in BMI, whereas patients with probable sarcopenia and moderate sarcopenia had higher BMI scores that reached overweight levels. This agrees with the increasing evidence of an association between sarcopenia and obesity, while at extreme levels of sarcopenia not only muscle mass but also fat mass seem to be reduced.

Furthermore, our study showed that elderly subjects with musculoskeletal disorders are also prone to malnutrition and sarcopenia and malnutrition in this population seemed to be related. In literature, it has been demonstrated that specific foods and dietary habits can help prevent the loss of strength and function that comes with age (25, 26). Several randomized controlled trials seem to indicate that dietary protein consumption is essential for avoiding sarcopenia and muscle loss (26). Both selenium and magnesium have been investigated as dietary supplements, and they seem to have a possible relationship with physical activity and muscular function in older people (26). Improving diet and nutrition may be useful for both the prevention and treatment of sarcopenia as low nutritional status is widespread in the elderly, especially in frail subjects (12). Therefore, a proper rehabilitation program for elderly subjects with both sarcopenia and musculoskeletal concerns should include not only motor and strengthening exercises, but also educational initiatives to promote an adequate nutritional status. Progressive elastic band resistance exercises have been demonstrated to reduce fat mass and to increase physical function in patients with sarcopenic obesity and sarcopenia (27–30). Both water- and land-based activities have been shown to be beneficial in maintaining strength and in improving lower-body flexibility, with aquatic exercise appearing a better activity to improve dynamic balance and to manage comorbidities (31–35). Nutritional interventions should also be involved in the rehabilitative protocols suggested to these patients. Therefore, the rehabilitation team should include a nutrition expert, in order to help the patients receive the appropriate amount of protein as principal anabolic stimuli for muscle protein synthesis (1.0–1.2 g/kg body weight per day) and all the supplements that support the musculoskeletal health, such as vitamin D, antioxidant nutrients and long-chain polyunsaturated fatty acids (25, 36).

In our study, arm and calf circumference were shown to be reduced especially in patients with severe sarcopenia. Therefore, this study supports the use of these measurements for aiding in sarcopenia detection, reconfirming Hu et al. and Kawakami et al. findings (22, 23). They could therefore represent rapid indicators for the assessment of patients at increased risk of developing sarcopenia and thus be employed in clinical practice. A significant direct correlation between BMI and MUAC and CC circumferences was also obtained in our study, in accordance with previous results (37). Additionally, a slender direct association between MNA and CC was found. As a result, CC could be employed as a rapid outpatient tool for the assessment of the elderly patient nutritional status.

Our results showed also a moderate but significant reduction in the physical status category of quality of life in subjects with probable sarcopenia and severe sarcopenia when compared with the group of patients who did not present sarcopenia. This agrees with previous literature in which an association between quality of life and nutritional condition was found (38, 39). It is well-known that sarcopenia increases the risk of physical limitation and disability, lowering patients’ quality of life.

According to our data, patients with both sarcopenia and worse nutritional status reported reduced quality of life. By reducing malnutrition and encouraging optimal functioning, good nutrition can enhance the health-related quality of life (38). Since low physical capacity and quality of life influence personal and social costs (40), it can be hypothesized that the development of programs aimed at preventing nutritional deficiencies in patients with musculoskeletal disorders could contribute not only to ameliorate quality of life but also to economic and social benefits.

From the findings of our study, we can conclude that evaluations of sarcopenia, quality of life, and nutritional status should become part of the rehabilitative outclinics protocol for elderly patients accessing the Rehabilitation Unit for musculoskeletal concerns. The management of elderly patients with sarcopenia or pre-sarcopenia conditions associated to musculoskeletal disorders should start with the early detection of different concerns. An early assessment resulting in the identification of otherwise neglected needs may contribute to the development of primary and secondary prevention strategies, avoiding the progression into real pathological conditions (i.e., probable sarcopenia into sarcopenia, nutritional deficiencies into malnutrition) (41). Subsequently, a multimodal and multidisciplinary rehabilitation program, including prevention strategies, motor activity, strengthening exercise, nutrition and educative interventions (42, 43), should be proposed.

The study has several limitations that should be taken into consideration when interpreting the results. Firstly, there is a potential for selection bias as the participant selection process may not accurately represent the broader population of elderly individuals with musculoskeletal disease. Secondly, the generalizability of the findings may be limited due to the nature of observational studies. The specific inclusion and exclusion criteria, as well as the characteristics of the participants, may restrict the ability to extend the results to broader populations or different settings. Thirdly, despite attempts to control for confounding through statistical analysis or study design, there may be unmeasured or unknown factors that influence the relationship between the variables under investigation.

## Conclusion

Real-life elderly subjects with musculoskeletal concerns are highly susceptible to sarcopenia. Therefore, rehabilitation for elderly patients with musculoskeletal concerns requires to be customized and multidisciplinary, addressing both the musculoskeletal condition and the needs associated to sarcopenia, including nutritional supplementation. To identify those who are more likely to develop sarcopenia, it is advisable to draw a user-friendly screening tool, e.g., using MUAC and CC. Nutritional assessment should be part of the screening process since early detection of a malnourished state may result in interventions to improve nutritional status and, as a result, quality of life. Future research should further investigate these aspects in order to enable the early identification of sarcopenia among elderly patients affected by musculoskeletal concerns and the formulation of customized multidisciplinary rehabilitative programs.

## Data availability statement

The raw data supporting the conclusions of this article will be made available by the authors, without undue reservation.

## Ethics statement

Ethical review and approval was not required for the study on human participants in accordance with the local legislation and institutional requirements. The patients/participants provided their written informed consent to participate in this study.

## Author contributions

Conceptualization and methodology, MM, DC, AF, YD and SM. Data collection, AB and MV. Data curation, MM, DC, AF, AB and NS. Data analysis, DC, AF and NS. Investigation, MM, DC, AB and MV. Writing—original draft preparation, MM and DC. Writing—review and editing, MM, DC, YD and SM. Supervision, SM. All authors contributed to the article and approved the submitted version.
